# On the Dressing of Wounds by Occlusion

**Published:** 1845-11

**Authors:** 


*On the Dressing of Wounds by Occlusion.*—For nearly three years, says M. Chassaignac, I have employed a mode of dressing wounds which I designate by the name of *dressing by occlusion*. In the case of a recent wound, I construct on the injured part a cuirass with adhesive plaster, divided into strips, which cover each other by imbrication. This covering is enclosed by a piece of linen spread with cerate, and pierced full of holes, then covered by lint kept in place by compresses and bandages. This dressing should remain for eight or ten days. If the suppuration be sufficiently free to require it, the external parts of the dressing, as far as the piece of linen spread with cerate inclusively, may be renewed, but the cuirass of adhesive plaster must not be interfered with.—*London Medical Times.*

